# The effects of heritage multilingualism on foreign language learning: a comparison of children with typical language development and developmental language disorder

**DOI:** 10.3389/fpsyg.2024.1521340

**Published:** 2025-01-15

**Authors:** Elena Tribushinina, Betül Boz

**Affiliations:** Institute for Language Sciences, Utrecht University, Utrecht, Netherlands

**Keywords:** multilingualism, English as a foreign language, developmental language disorder, out-of-school exposure, cross-language relationships, bilingual advantages

## Abstract

**Introduction:**

Both multilingualism and developmental language disorder (DLD) may be associated with inferior performance in the majority language, albeit for different reasons. At the same time, there is a growing body of evidence that multilingualism may have a positive effect on foreign language performance. This study tests the hypothesis that the positive effects of multilingualism on foreign language learning may be smaller in children with DLD compared to their multilingual peers with typical language development.

**Methods:**

In a 2 × 2 design, we compare the effects of multilingualism and DLD on English as a foreign language performance and majority language performance of multilinguals and monolinguals with and without DLD. The participants were primary school children (aged 9–13) acquiring Dutch as the majority language and learning English as a school subject. English skills were measured with a vocabulary test, a grammar test and the Multilingual Assessment Instrument for Narratives (MAIN). Dutch skills were assessed with the Litmus Sentence Repetition Task and the MAIN task. The MAIN narratives in both languages were analyzed for fluency, lexical diversity, syntactic complexity and grammatical accuracy. The control variables included age, working memory, declarative memory, procedural memory and (for English) amount of extracurricular exposure and length of instruction. Data were analyzed by means of multilevel linear regression.

**Results:**

The results demonstrate that both multilingualism and DLD were associated with lower scores on the Dutch Sentence Repetition Task and lower grammatical accuracy of narratives. In English, the multilinguals outperformed monolinguals on all measures, except grammatical accuracy of narratives, and the interactions between Background and Group were not significant. Another strong predictor of EFL performance, along with the multilingual status, was extracurricular exposure to English.

## Introduction

About half of the world’s children grow up with two or more languages from birth (De Houwer, 2014). Although “bilingualism” has often been used as an umbrella term for the acquisition of two or more languages, there has been a recent shift toward a more frequent use of the umbrella term “multilingualism” to emphasize that heritage bilinguals often develop multilingual competence through contact with more than two languages, supported by findings demonstrating that third language (L3) acquisition is qualitatively different from second language (L2) acquisition (e.g., Pliatsikas et al., 2020; Yee et al., 2023). In this paper, we will use the term “multilingual” to refer to children who grow up with a majority language (ML) and with one or more heritage languages (HLs). Multilingual children often lag behind their monolingual peers in the ML (which is also the school language) and heavy-verbal subjects but tend to outperform monolinguals when it comes to foreign languages (FLs) (Hopp et al., 2019; Chachashvili-Bolotin and Kreiner, 2022; Tribushinina and Mackaaij, 2023). Such benefits of multilingualism in FL learning are known as “bilingual FL learning advantages” or “L3 advantages.” The term “bilingual advantage” has been recently criticized in the literature on cognitive effects of multilingualism as being too simplistic. Researchers started using the terms “bilingual effects” and “bilingual adaptations” instead, reflecting the capacity of the human brain to adapt to the more complex linguistic environment in multilingual contexts (Bialystok, 2017; D’Souza and D’Souza, 2021; Festman et al., 2023; Leivada et al., 2021; Masullo et al., 2024; Pliatsikas et al., 2020; Yee et al., 2023). Following this trend, the present paper will use the terms “multilingual effects” and “multilingual adaptations” in the comparisons of FL learning ability of monolingual and multilingual children (cf. Festman, 2021).

Effects of multilingualism on learning FLs, mainly English as a foreign language (EFL), were initially studied in bilingual communities where both the minority language and the ML are supported in education (Swain et al., 1990; Cenoz and Valencia, 1994; Lasagabaster, 2000; Muñoz, 2000; Sanz, 2000; Sagasta Errasti, 2003; Mady, 2017; Agustín-Llach, 2019). These studies revealed that bilingual pupils tend to outperform their monolingual peers on EFL skills. More recently, differences between multilinguals and monolinguals in (E)FL learning have also been targeted in immigrant contexts, where children acquire a HL along with the ML. The evidence from this line of research is mixed: While a growing number of studies indicate that multilingual pupils outperform their monolingual peers on FL learning at school (Schwartz et al., 2007; Abu-Rabia and Sanitsky, 2010; Rauch et al., 2011; Kopečková, 2016; Maluch and Kempert, 2017; Maluch et al., 2015, 2016; Hopp et al., 2019; Nguyen and Winsler, 2021; Chachashvili-Bolotin and Kreiner, 2022; Geiss et al., 2022), there are also studies that found no differences between multilinguals and monolinguals on FL measures (Schoonen et al., 2002; Edele et al., 2018; Festman, 2018; Lorenz et al., 2020, 2023, 2024). There are even studies in which monolinguals outperformed their multilingual peers on EFL proficiency (Van Gelderen et al., 2003; Lorenz et al., 2024).

So far, the effects of multilingualism on FL learning have been mainly addressed in children with typical language development (TLD). However, three recent studies extended this line of research to learners with developmental language disorder (DLD), one of the most common learning disabilities affecting about 7–8% of children (Bishop, 2017). DLD is a neurodevelopmental condition that involves deficits in the understanding and production of language, which cannot be attributed to factors such as lower intelligence, hearing deficits, mental or physical disability, emotional disorder or environmental deprivation (Leonard, 2014; Bishop, 2017). Research on (E)FL learning by children with DLD is only emerging. In the very first publication on this topic, Zoutenbier and Zwitserlood (2019) compared reading and listening EFL skills of Dutch primary school pupils with DLD to age-related norms for typically-developing children. The majority of the participants had low percentile scores ranging from 1 to 20. This was also the first study to compare EFL skills of monolingual and multilingual children with DLD. No effects of multilingualism were found.

This new line of research was continued by Tribushinina and Mackaaij (2023), which was also the first study to include measures of out-of-school exposure to English in comparing EFL skills of monolingual and multilingual pupils. The results revealed that without controlling for amount of extracurricular exposure to English, multilingual primary school children with DLD outperformed their monolingual peers with DLD on seven of the eight EFL measures included in the study. However, the multilingual group also had more exposure to English outside of the classroom, mainly through watching videos, gaming and listening to English songs. When controlling for differences in out-of-school exposure, three of the initially significant differences became non-significant, but the positive effects of multilingualism were still visible in English vocabulary, grammar and syntactic complexity of narratives. Interestingly, despite their superior performance in the FL (English), the multilingual group was outperformed by their monolingual peers in the ML (Dutch). The intriguing asymmetry between superior EFL performance and inferior ML performance is in line with prior research on multilingual children with TLD demonstrating a similar FL-ML mismatch (Hopp et al., 2019; Chachashvili-Bolotin and Kreiner, 2022).

Stolvoort et al. (2024) also compared English vocabulary and grammar skills of multilingual and monolingual pupils with DLD, and controlled for differences in the amount of exposure to English outside of the classroom. Unlike the results of Tribushinina and Mackaaij (2023) with primary school children, this study, conducted with secondary school pupils, revealed no significant differences between multilingual and monolingual children with DLD on EFL vocabulary and grammar. The lack of the significant effects might have been due to the small sample size (5 monolinguals and 13 multilinguals) used in Stolvoort et al. (2024). However, it might also be the case that multilingual effects on FL learning become weaker with age (Maluch et al., 2016; Hopp et al., 2019).

In summary, our understanding of the effects of multilingualism on FL learning by children with DLD is still very limited, and the findings are mixed. The present study continues this line of research by comparing EFL skills of multilingual and monolingual children with DLD in the upper grades of primary education. The main novelty of this research is that we study the presence and compare the extent of multilingual effects in FL learning in children with DLD and TLD. As explained in more detail below, there are reasons to expect that multilingual adaptations could be less strong in children with DLD compared to typically-developing peers. Like Tribushinina and Mackaaij (2023), we carefully control for differences in extracurricular exposure to English to rule out the possibility that any group differences are due to multilinguals’ having either more (Tribushinina and Mackaaij, 2023) or less (Tribushinina et al., 2024a) exposure to English outside of the classroom. Unlike prior studies on the topic, we also control for verbal working memory, declarative memory and procedural memory because these memory systems play a crucial role in language learning and may reveal differential effects of multilingualism and DLD.

### Potential sources of multilingual effects in FL learning

Positive effects of multilingualism on FL learning have been attributed to four potential sources: (i) positive transfer of language knowledge from more than one language, (ii) more developed metalinguistic awareness, (iii) enhanced executive functions, and (iv) more positive attitudes toward language learning (see reviews in Cenoz, 2003; Hirosh and Degani, 2018; Festman, 2021). Although it is beyond the scope of this paper to verify the role of these four factors as mediators of multilingual effects on FL learning, the differential profiles of children with and without DLD on these four dimensions are informative for making predictions regarding the extent of multilingual adaptations in children with TLD and DLD.

The first potential source of differences between multilingual and monolingual FL learners is the fact that multilinguals have knowledge of more than one lexicon and grammar to transfer to a new language. Positive transfer is more likely to take place if the languages in the multilingual mind are typologically similar (e.g., Blom and Paradis, 2013, 2015) but transfer from typologically remote languages has also been reported (e.g., Siu and Ho, 2015). Examples of positive transfer in the domain of vocabulary include cognate facilitation effects, entailing that cognates are easier to learn and to retain than non-cognates. Multilinguals may recognize more cognates in the L3 due to having two or more lexicons to draw upon. For example, even though English and Dutch, both West-Germanic languages, share a lot of cognates, Turkish-Dutch learners of English may have an additional advantage because some of the English cognates are reinforced by both their languages (e.g., *salmon-zalm-somon*) and some Turkish-English cognates (e.g., *barrier–bariyer; sausage–sosis*) are not cognates with Dutch. An example of positive transfer in the grammatical domain can be found in the study by Kolb et al. (2022). They compared grammaticality judgments of Russian-German bilinguals to those of Russian and German monolinguals and found positive transfer from both Russian and German to L3 English. For structures that were similar between Russian and English (e.g., adverb placement), the bilinguals performed worse than Russian monolinguals, but better than German monolinguals. For the structures that were more similar between German and English (e.g., determiner use), the opposite pattern was found.

Thus far, it is not clear whether positive cross-language transfer is available to children with DLD. To the best of our knowledge, no studies have targeted positive transfer in L3 learners with DLD, but there is some relevant research on L2 acquisition. For example, some studies including participants with DLD demonstrate positive transfer between language-control skills (e.g., rapid automatic naming), but lack of transfer in knowledge-related tasks (e.g., vocabulary, grammar) (Verhoeven et al., 2012; Ebert et al., 2014). On the other hand, there is evidence that children with DLD benefit from cognate relationships between L1 and L2 (Payesteh and Pham, 2022; Tribushinina et al., 2023), to the same extent as their peers with TLD (Kohnert et al., 2004; Grasso et al., 2018).

For grammar, the evidence is scarce and more mixed. Blom and Paradis (2013) report that both children with and without DLD are more successful in acquiring L2 English verb morphology if they speak an inflectional (rather than an isolating) L1, and this effect is similar in children with and without DLD. On the contrary, Blom and Paradis (2015) demonstrate that the effects of positive transfer in the domain of verbal morphology were mainly driven by the TLD group and suggest that children with DLD may be less able to spontaneously instantiate positive transfer in the domain of grammar. The findings reported by Tribushinina et al. (2020) seem to confirm this idea. In their longitudinal study, L1 Russian vocabulary predicted EFL vocabulary size in children with and without DLD. However, L1 grammar only predicted EFL grammar skills in the TLD group (after 1 year of English lessons); at none of the measurements did children with DLD show any significant relationships between L1 and EFL grammar. The authors proposed that DLD may selectively impair positive cross-language transfer in the domain of morphosyntax, which is known to be the area of core difficulty in DLD (Leonard, 2014). If positive transfer is (selectively) unavailable in DLD, multilinguals with DLD may benefit less from their prior language knowledge in acquiring an L3. Hence, the effects of multilingualism on FL learning might be smaller for children with DLD compared to their peers with TLD.

Differences between monolinguals and multilinguals in FL performance have also been ascribed to enhanced metalinguistic awareness associated with multilingualism. Metalinguistic awareness involves the ability to view language as a system, to compare languages and to reflect on language structure. A growing body of evidence demonstrates a positive relationship between multilingualism and metalinguistic awareness (Bialystok, 2001; Dolas et al., 2022; Hofer and Jessner, 2019; Jessner, 2006; Foursha-Stevenson and Nicoladis, 2011; Kuo and Anderson, 2010). Multilingual children compare their languages from early on and develop insights into how languages work. Metalinguistic awareness has been shown to positively predict novel language learning success (e.g., Hopp et al., 2019; Tribushinina et al., 2021; D’Angelo and Sorace, 2022). Therefore, it can be assumed that the effects of multilingualism on FL learning may be explained by differences in metalinguistic awareness (Rauch et al., 2011).

Metalinguistic abilities of children with DLD have received relatively little attention in the literature. To the best of our knowledge, Kamhi and Koenig (1985) is the only study comparing metalinguistic awareness of children with and without DLD. Their results show that children with DLD had no difficulty recognizing phonological and semantic violations but performed significantly worse than typically-developing controls on recognizing ungrammaticalities, once again reinforcing the vulnerable status of morphosyntax in DLD. To date, it is not clear whether the positive effects of multilingualism on metalinguistic awareness, often attested in children with TLD, also hold for children with DLD, due to paucity of available studies and lack of uniform operationalizations of metalinguistic awareness. For example, Peristeri et al. (2019) used a word definition task as a measure of metalinguistic awareness and found that multilinguals with DLD outperformed monolinguals with DLD. In contrast, Tribushinina and Mackaaij (2023) found no differences between monolinguals and multilinguals on an English grammaticality judgment task (a metalinguistic measure) even though multilinguals outperformed monolinguals on the English grammar test (a linguistic measure). Hence, the evidence is inconclusive and more research is clearly warranted. In light of the findings reported by Kamhi and Koenig (1985), it may be assumed that children with DLD are less able than their peers with TLD to draw on metalinguistic awareness, which may reduce the positive effects of multilingualism on FL learning.

Superior performance of multilinguals on FL learning has also been attributed to positive effects of multilingualism on executive functions. Although the findings are highly controversial (e.g., Duñabeitia et al., 2014; Paap et al., 2015; Scaltritti et al., 2015; Blom et al., 2017), there is a growing body of evidence that under some circumstances (some aspects of) multilingual experience may have a positive effect on, for example, selective attention (e.g., Blom et al., 2017; Olguin et al., 2019), inhibition (Carlson and Meltzoff, 2008; Engel de Abreu et al., 2012; Kroll and Bialystok, 2013; Nguyen et al., 2024), cognitive flexibility (Kovács and Mehler, 2009; Comishen et al., 2019; Nguyen et al., 2024) and working memory (Blom et al., 2014; Morales et al., 2013; Blom and Boerma, 2017; Durand López, 2021). Although DLD appears to have a negative effect on executive functioning (Blom and Boerma, 2017; Ebert et al., 2019; Park et al., 2020; Ebert, 2021), multilinguals with DLD have been reported to outperform monolinguals with DLD in verbal working memory (Blom and Boerma, 2017), allocation of attention (Park et al., 2019) and processing speed (Ebert, 2021). However, some studies report no positive effects of multilingualism on executive functioning. For instance, Ebert et al. (2019) demonstrate that 6- to 8-year-old children with DLD score lower than children with TLD on attentional control and sustained attention, but no differences were found between English monolinguals and English-Spanish bilinguals. Assuming that executive functions are involved in learning new languages (Shokrkon and Nicoladis, 2022), multilinguals may experience additional advantages in FL learning through enhanced cognitive skills.

Another potential source of multilingual effects in FL learning lies in the affective domain. Although evidence is limited to adults and secondary school children with TLD, it has been suggested that multilinguals may show greater interest in FLs (Merisuo-Storm, 2007; Nguyen and Winsler, 2021) and experience less anxiety using FLs (Botes et al., 2020; Dewaele and MacIntyre, 2014; Rutgers and Evans, 2017; Thompson and Lee, 2013). So far, only one study has scrutinized the relationship between attitudes and FL learning success of children with DLD. Stolvoort et al. (2024) report that EFL anxiety is negatively related to grammar and vocabulary outcomes of secondary school pupils with DLD in special education, whereas attitudes toward English lessons positively predict performance, replicating the pattern repeatedly found for learners with TLD (e.g., Sanz, 2000; Leona et al., 2021). We may cautiously assume that positive effects of multilingualism on language learning attitudes may also support FL learning by multilingual pupils with DLD.

In summary, for three of the four potential sources underlying positive effects of multilingualism on novel language learning (transfer, metalinguistic awareness, executive functions), it has been shown that they are negatively affected by DLD. Therefore, it is plausible to assume that positive effects of multilingualism on FL learning might be less strong in children with DLD.

### Memory systems in DLD and multilingualism

FL learning is supported by different memory systems. Working memory is known to be heavily involved in language acquisition in general and in FL learning in particular (Baddeley, 2003). Kormos and Sáfár (2008) demonstrate that working memory strongly correlates with a variety of EFL skills (reading, listening, speaking, vocabulary and grammar) in adolescent pupils. Similarly, Engel de Abreu and Gathercole (2012) demonstrate that verbal working memory predicted grammar skills in L1, L2 and L3 of Luxembourgian children acquiring L2 German and L3 French in a classroom setting. A similar relationship between grammar learning and verbal working memory was demonstrated for children acquiring an L2 in a naturalistic setting (Verhagen and Leseman, 2016). Multilingualism and DLD seem to affect working memory in differential directions. DLD is associated with deficits in working memory (e.g., Blom and Boerma, 2017), whereas multilingualism has been shown to have a positive effect on working memory (e.g., Blom et al., 2014; Morales et al., 2013; Blom and Boerma, 2017; Durand López, 2021), even though the effects appear small (Monnier et al., 2022) and evidence is still very limited (Giovannoli et al., 2020).

L2 learning in general and instructed FL learning in particular also rely on declarative and procedural memory. Procedural memory subsumes the acquisition of rule-based aspects of language (syntax, regular morphology, partly phonology), whereas declarative memory serves the acquisition of vocabulary, irregular morphology and explicit grammar rules (Ullman and Pierpont, 2005). Although most of grammar should be acquired through procedural memory in naturalistic L2 acquisition (and in FL acquisition from informal out-of-school exposure), classroom-based FL learning containing explicit grammar instruction also involves grammar learning through declarative memory. The Procedural Deficit Hypothesis (Ullman and Pierpont, 2005) posits that procedural memory of individuals with DLD shows structural abnormalities, which renders regular morphosyntax the area of particular difficulty. In contrast, the declarative memory system is suggested to be spared in DLD and to take over some of the functions of the malfunctioning procedural memory. Along these lines, Lum et al. (2012) report that, in children with TLD, declarative memory predicts lexical learning and procedural memory is related to grammar outcomes, whereas in children with DLD declarative memory predicts both lexical and grammatical acquisition. This said, there is also evidence against the Procedural Deficit Hypothesis (see a meta-analysis in Lammertink et al., 2020). For example, Jackson et al. (2020) found negative effects of DLD on working memory, declarative memory, and procedural memory in 5- to 8-year-old children. However, the difference between children with and without DLD on verbal declarative and procedural memory became non-significant once verbal working memory was controlled for. In sum, there appears to be a consensus that declarative memory is not affected by DLD, but it is still a matter of debate whether DLD involves procedural learning deficits.

There is also a lot of controversy regarding the impact of multilingual experience on declarative and procedural memory. Brito and Barr (2012) report positive effects of bilingualism on (declarative) memory generalization in infancy. Durand López (2021) found that multilinguals outperformed monolinguals on working memory tasks, but not on semantic memory (subcomponent of declarative memory). In contrast, de Jesus (2012) found that adult multilinguals outperformed monolinguals in (verbal and non-verbal) declarative memory tasks. In a longitudinal study spanning throughout adulthood, Ljungberg et al. (2013) show that Swedish L1 speakers who were more proficient in an L2 (usually English) outperformed a group categorized as Swedish monolinguals on several verbal tasks measuring episodic memory, a subdomain of declarative memory. However, since only verbal tasks were used, the directionality of the effects in this study is not clear.

Regarding procedural memory, evidence is also scarce and mixed (see a review in Bulgarelli et al., 2018). de Jesus (2012) reports that, in an adult sample, the positive effects of multilingualism were limited to the speed of responses in a non-verbal task. However, multilingual children (De Bree et al., 2017) and adults (Nation and McLaughlin, 1986) were shown to be more successful than monolinguals on artificial grammar learning tasks under implicit learning conditions. Benefits of bilingual experience for statistical learning in adulthood have been demonstrated by Wang and Saffran (2014). In contrast, Poepsel and Weiss (2016) and Aguasvivas et al. (2024) found no overall advantages of multilingualism in statistical learning in adults. The latter study suggested that bilingual effects in FL learning may be limited to the lexical domain. To the best of our knowledge, only one study has compared procedural learning abilities of monolingual and multilingual children with DLD (Park et al., 2018). The children with DLD did not exhibit learning of sequential patterns, in contrast to the children with TLD, but no difference was found with regards to multilingual background.

To recapitulate, procedural, declarative and working memory are heavily involved in FL learning and may show differential effects of multilingualism and DLD. Therefore, we will control for declarative, procedural and working memory capacity in our analyses.

## The present study

This study builds on earlier research by comparing FL skills of monolinguals and multilinguals with DLD, while controlling for differences in out-of-school exposure. The study was conducted in the Netherlands, where children (with and without DLD) start learning English as a school subject in primary school. We use the term “multilingual” with reference to children whose parents speak one or more HL at home (Dutch). For brevity and convenience, the term “monolingual” is used with reference to children who were raised in the ML (Dutch) only, at the same time acknowledging the fact that these children also have a certain degree of EFL competence (like everybody in the Netherlands). Unlike prior studies on this topic, our research compares the extent of multilingual effects in children with DLD and peers with TLD. Our study is also the first to control for differences in procedural, declarative and working memory in comparisons of EFL skills of multilingual and monolingual learners. To the best of our knowledge, only one previous study (Hopp et al., 2019) included working memory as a control variable in comparing EFL skills of multilingual and monolingual children and showed that verbal working memory predicted both vocabulary and grammar scores. We also aim to replicate the intriguing finding from previous research demonstrating that the same multilingual children may lag behind in the ML but excel in EFL (Hopp et al., 2019; Tribushinina and Mackaaij, 2023). The following research questions are addressed:

1. Do multilinguals outperform monolinguals on EFL proficiency? And if so, is this effect stronger in the TLD group?
2. Do multilingualism and DLD negatively affect performance in the ML (Dutch)? And if so, is the gap between multilinguals and monolinguals larger in the DLD group?

Following the growing body of studies demonstrating that multilinguals outperform monolinguals on FL skills (see Introduction), we predicted that our multilingual participants would have higher EFL scores compared to their monolingual peers, even after controlling for differences in out-of-school exposure to English (Tribushinina and Mackaaij, 2023; Tribushinina et al., 2024a). Since DLD has been shown to negatively affect metalinguistic awareness (Kamhi and Koenig, 1985), executive functions (Blom and Boerma, 2017; Ebert et al., 2019; Park et al., 2020; Ebert, 2021) and positive transfer (Blom and Paradis, 2015; Tribushinina et al., 2020), we hypothesized that the extent of this effect would be smaller in the DLD group, which should manifest in a significant interaction between Group (DLD; TLD) and Background (monolingual; multilingual).

Both multilingualism and DLD are expected to have a negative effect on ML (Dutch) scores. Multilingual children have reduced exposure to each of their languages because their time is divided between two or more languages, which may affect the acquisition of vocabulary and frequency-sensitive grammatical phenomena (e.g., Driessen et al., 2002; Foursha-Stevenson and Nicoladis, 2011; Nicoladis and Marchak, 2011; Unsworth et al., 2014). DLD is associated with reduced intake, and children with DLD need more time and exposure to acquire the same phenomena that children without DLD acquire with less input (Tomblin et al., 2007; Evans et al., 2009). The cumulative effects hypothesis predicts that the gap between monolingual and multilingual children should be larger in the DLD group (Paradis, 2010; Paradis et al., 2017). However, the bulk of research evidence shows that the effects of reduced exposure due to multilingualism are not different in children with DLD (see review in Novogrodsky and Meir, 2020). Therefore, we did not expect to find a significant interaction between Group and Background for the ML (Dutch).

## Method

### Participants

Seventy-five children were recruited in the final three grades of primary (special) education (grades 4–6): 20 monolinguals with TLD, 18 multilinguals with TLD, 24 monolinguals with DLD and 13 multilinguals with DLD (see Table 1). All participants with DLD were recruited from the same primary special school (cluster-2 school, for children with language disorders and hearing impairments) in the South of the Netherlands. These participants had been independently diagnosed with DLD following a standardized protocol, which requires an overall score of at least 2 *SD* below age-appropriate norms on a standardized Dutch language test or scores of at least 1.5 *SD* below the age-appropriate mean score on at least two of the four subscales of a standardized language test (Stichting Siméa, 2014). A hearing impairment and intellectual disability constitute exclusion criteria. Participants with TLD attended regular primary schools (*n* = 4) in the central and eastern parts of the Netherlands. All participating schools had similar SES levels (low-to mid-SES), as reported by school management.

**Table 1:** Participant characteristics.

Group	Background	*n*	Mean age	Age range	Sex	n 4th grade	n 5th grade	n 6th grade
TLD	Monolingual	20	10;3	8;8–11;7	7 M, 13F	9	2	3
	Multilingual	18	11;0	9;1–11;11	11 M, 7F	3	6	9
DLD	Monolingual	24	11;1	9;4–12;6	15 M, 9F	7	7	10
	Multilingual	13	10;10	9;7–13;2	12 M, 1F	5	7	1

All monolingual participants were raised only in the ML (Dutch), their parents did not use any other language in communication with their children even though all children were exposed to some English at home (mainly through media). The multilingual participants spoke Dutch as the ML and also received parental input in and spoke at least one HL with their parents. We followed Kohnert’s (2010) definition of multilinguals as “individuals who receive regular input in two or more languages during the most dynamic period of communication development – somewhere between birth and adolescence” (p. 457). The languages spoken by the multilingual group with TLD included Turkish (*n* = 9), Russian (*n* = 2), Moroccan/Arabic (*n* = 2), both Italian and Moroccan (*n* = 1), Kurdish (*n* = 1), Bosnian (*n* = 1), Somali (*n* = 1), and Berber (*n* = 1). The backgrounds of the multilinguals with DLD included Turkish (*n* = 6), Turkish and Greek (*n* = 1), Turkish and Arabic (*n* = 2), Polish (*n* = 2), Moroccan (*n* = 1), Thai (*n* = 1), and Limburgisch (*n* = 1). Unfortunately, we could not administer parental questionnaires and obtain information on children’s exposure to and proficiency in the HL(s). This study was conducted in the school year 2020–2021, during the Covid pandemic, when the parents were not allowed on the school premises. The special school did not allow us to contact the parents in order not to overburden them even more during this challenging period. Hence, we can only compare children who did (multilinguals) and did not (monolinguals) hear and speak a HL at home, without exploring more fine-grained effects of levels of bilingual proficiency (e.g., Lasagabaster, 2000; Muñoz, 2000; Sagasta Errasti, 2003; Edele et al., 2018). For the same reason, we could not collect individual information on parental education, which is a limitation. However, we tried to match the mainstream schools to the socio-economic level of the special school for children with DLD (mainly low-to mid-SES families, as reported by the schools).

Overall, there was no age difference between monolinguals and multilinguals [*t*(73) = −1.25, *p* = 0.216] and between children with and without DLD [*t*(73) = 1.77, *p* = 0.080]. Monolinguals and multilinguals with DLD did not differ in age [*t*(35) = 0.61, *p* = 0.544], but the multilingual group with TLD was older than the monolingual group with TLD [*t*(36) = −2.99, *p* = 0.005]. Therefore, age will be included as a control variable in all the analyses.

All children were learning English as a school subject (30–45 min a week). Mainstream primary schools in the Netherlands teach English implicitly (i.e., without explanation of grammar rules) and focus on communicative competences. In contrast, English instruction at the special education school that participated in our study was mostly explicit, following a multisensorial CodeTaal method that was specifically designed for pupils with DLD (Tribushinina et al., 2022).

Dutch primary schools are obliged to start English classes in the 5^th^ grade at the latest (i.e., around age 10) but are free to introduce English instruction in the lower grades (as early as kindergarten). Therefore, the schools in our sample differed in the onset of the English lessons. Furthermore, within the same schools, the children in the higher grades (5–6) had received more English instruction than the participants in the younger grades. To control for this variability in the amount of EFL instruction, we calculated Length of EFL Instruction for each participant by taking the sum of the total of months from the first moment of instruction until the first moment of testing. Weeks and months in which schools were on a break or in lockdown were not excluded from the count, because those numbers were the same for all participants. The differences between multilinguals and monolinguals were not significant in either group [*t*_TLD_ (36) = 1.86, *p*_TLD_ = *0*.071; *t*_DLD_ (35) = 1.92, *p*_DLD_ = 0.063]. However, they approached significance and there was a lot of intra-group variation (Table 2). To control for this variability, Length of EFL Instruction was included as a control variable in all analyses of the English data.

**Table 2:** Means (and standard deviations) of the background measures.

	DLD		TLD	
	Multilingual	Monolingual	Multilingual	Monolingual
Exposure (max. 28)	12.54 (3.23)	11.46 (5.57)	15.67 (6.00)	12.80 (5.09)
Length of EFL instruction (in months)	11.69 (7.57)	18.00 (10.42)	12.67 (8.03)	20.25 (15.52)
Procedural memory (max. 16)	9.15 (2.30)	9.5 (2.3)	10.11 (2.03)	9.20 (2.95)
Declarative memory (max. 40)	32.62 (5.17)	35.8 (3.3)	35.56 (3.62)	35.65 (3.68)
Working memory (max. 80)	30.92 (12.95)	36.6 (10.2)	44.56 (9.66)	43.40 (10.16)

### Test instruments and procedure

Our focus was on EFL performance of monolingual and multilingual children. Therefore, we used an extensive test battery in English. However, we also needed a measure of their proficiency in the ML to rule out the possibility that the multilingual sample happened to contain stronger language learners overall. We did not strive for parallel tests because Dutch and English have a very different status in the Netherlands and in the participants’ lives (ML *vs*. FL). The Dutch measures were not meant to study the workings of cross-language transfer, as this falls beyond the scope of our research. This being said, calculating correlations between Dutch and English measures can shed further light on the ability of children with DLD to use positive transfer in learning a new language.

#### English vocabulary task

To measure English vocabulary, we used a receptive vocabulary task developed by Tribushinina and Mackaaij (2023) for research on DLD learners of English in classroom settings. This test was reckoned more suitable for our purposes than standardized tests such as PPVT because standardized vocabulary tests were designed for children acquiring English as L1 with plenty of exposure rather than for FL learners in classroom settings. One obvious problem with PPVT is that cognates are unevenly distributed across sets. Furthermore, the intended levels of complexity verified for native speakers do not hold in EFL contexts (see Tribushinina et al., 2020 for a more detailed discussion).

Our test measured the ability to translate 40 English words to Dutch: 16 nouns, 12 adjectives, 10 verbs, and 2 prepositions (*α* = 0.94). The words were selected based on the wordlists for upper grades of primary school in the Netherlands [e.g., a wordlist on Wozzol (2020) for the 4th grade]. Half of the words within each word class were Dutch-English cognates, and the other half were non-cognates. The cognates and the noncognates were matched on frequency and word length in both English and Dutch. A PowerPoint was used to administer the test. Each slide showed one written English word, while a recording of the oral production of that word was played. The test was preceded by three practice items.

The participants were told that they were going to translate English words to Dutch, and that they would only get one opportunity to see and listen to the words they would translate. Participants with TLD took the test in groups, with a maximum of nine people at a time. They were shown the PowerPoint on a big screen at the front of the classroom, or on a laptop screen (with a maximum of three people at a time). They were given the answer sheet to write down the answers. The participants with DLD were tested individually using a laptop. After they gave a translation of the English word, the researcher administering the test would write down their answer on the answer sheet. This was done to lower the task demands because DLD has a high comorbidity with dyslexia (Snowling et al., 2020), even though we did not have the exact information on the comorbid disorders in our sample. It took the participants about 15 min to complete the task.

One point was awarded for each correct answer, leading up to a maximum of 40 points. Only full points were awarded, and spelling errors did not detract from the awarded point for a correct translation. Furthermore, a point was awarded if a translation was substituted by a correct description of the meaning of the word or if the word was acted out, since this indicates that the participant did know what the word means and how to use it. For instance, a point was awarded for the word *sneeze* when it was translated as *niezen* ‘sneeze’, *dat doe je wanneer je aan peper of stof ruikt* ‘this is what you do if you smell pepper or dust’, or *hatsjoe!* (onomatopoeia).

#### English grammar test

Grammar knowledge was tested with a paper-and-pencil grammar test developed by Tribushinina and Mackaaij (2023), based on the Pearson Longman English Language Placement Test. This test was included because it allowed us to assess the participants’ productive grammar knowledge using a test format that is commonly used in (E)FL classes at school. The test included 30 English sentences with a gap (α = 0.87). Four multiple-choice options were given to fill in each gap. The target constructions included verb and noun morphology, prepositions, word order and negations. The participants were given 20 min to complete the test, but most of them finished within 10–15 min. One point could be scored for each correct answer, leading up to a maximum of 30 points.

#### Narrative task (English and Dutch)

To assess the children’s oral skills in English (FL) and Dutch (ML), we elicited narratives using the Multilingual Assessment Instrument for Narratives (MAIN) (Gagarina et al., 2012). This instrument was developed for research with multilingual populations to measure narrative ability in different languages using parallel picture stories, matched for the number of protagonists, episodes, and episodic complexity. In this study, we used the Dog Story and the Cat Story, both consisting of six pictures. Half of the participants produced the Cat Story in Dutch and the Dog Story in English, and for the other half this was reversed. The first story was to be told in Dutch and the second story in English to allow the participants to familiarize themselves with the task in the ML before attempting it in English (FL) because their proficiency in English was more limited.

The participants were all tested individually in a quiet room at their schools following the procedure outlined in the MAIN manual (Gagarina et al., 2012). The participants were allowed to scrutinize all six images before they were instructed to tell the story, two pictures at a time. It often happened that the participants did not know a word they wanted to use, especially in English. In those cases, participants were told to try anyway, by, for example, making up a word or resorting to Dutch.

The stories were transcribed using CLAN (MacWhinney, 2000). The transcribed utterances were transformed into C-units (communication units) following the procedure described by Tribushinina and Mackaaij (2023). C-units are defined as independent clauses, consisting of a subject-verb proposition, and all of its modifiers (Curenton, 2004).

The narratives were analyzed for fluency, lexical diversity, syntactic complexity and accuracy (Housen and Kuiken, 2009). *Fluency* was operationalized as the number of word tokens in a narrative. *Lexical diversity* was measured by counting the total number of word types in a narrative. *Syntactic complexity* was operationalized as a mean length of C-unit (MLCU), which was calculated by dividing the number of words by the number of C-units within one transcription. *Accuracy* was measured as a number of grammatical mistakes divided by the number of words in a narrative.

#### Sentence repetition task (Dutch)

The Litmus Sentence Repetition Task (SRT) (Marinis and Armon-Lotem, 2015) was used to measure the participants’ proficiency in Dutch. Through piloting, we established that the English version of the task was too difficult for our EFL learners. The rationale of the test is that the sentences are too long to allow passive parroting. The Litmus SRT is considered an appropriate instrument for measuring language proficiency at multiple levels, including lexical knowledge, parsing of spoken sentences, speech production and, particularly, grammatical ability (Klem et al., 2014; Polišenská et al., 2014).

The test consisted of 30 Dutch sentences and two practice trials. The test was administered to one participant at a time by means of a PowerPoint with prerecorded sentences produced by a female voice. The task was designed as a treasure hunt. The participants were instructed that they were only allowed to listen once to each sentence and to repeat it verbatim, or finish it in the way they thought it might have finished in case they did not remember.

Litmus SRTs can be scored in different ways. This study included three scoring mechanisms, Score 0–3, Grammaticality, and Target Structure (Marinis and Armon-Lotem, 2015). Since all the scoring schemes produced the same results in the statistical analyses, we will only report the scores based on the 0–3 scheme. By this method, a score of 3 points was awarded if there were 0 changes between model sentence and the participant’s response. Two points were given if there was only one change, 1 point if there were two or three changes, and 0 points in case of four or more changes. The maximum possible score was 90.

#### Declarative memory test

To assess the participants’ declarative memory, we used an adapted version of the Recognition Memory after Incidental Encoding (RMIE) task developed by Hedenius et al. (2013). The test consisted of two phases, with a one-hour break between them. In the encoding phase, the participants saw 40 black and white pictures, including 20 pictures of real objects and 20 of made-up objects. Their task was to indicate whether the objects were real. In the recognition phase, they also saw 40 pictures, including 20 pictures of familiar objects (10 real, 10 made-up) that had also appeared in the encoding phase, along with 20 new pictures (half real). In this phase, the participants were to indicate whether they had seen the picture in the first phase.

The pictures were presented using PowerPoint presentations. The pictures of real and made-up objects were shown in a pseudo-randomized order. In both phases, the real and made-up pictures did not appear more than three times in a row. Likewise, in the recognition phase, the pictures that were seen in the encoding phase and the pictures that were newly added did not appear more than three times in a row either. One picture per slide would appear within a time frame of 1,500 milliseconds (ms), after which an empty slide with only the number of the picture would be shown for 4,500 ms, during which participants could respond.

The test was administered in small groups of maximally 10 children at a time using a big screen in front of the classroom. The participants were given response sheets on which they could indicate whether the pictures showed real or made-up objects (encoding phase) and whether they had seen the pictures in the first session (recognition phase). It took the participants 4 min to finish each task/phase, excluding the training trials and explanation of the test.

The declarative memory score was calculated as a sum of correct responses (1 point per correct response) with a maximum of 40 points. The scores of the encoding phase were only used to make sure that participants paid attention to the pictures. None of the participants were excluded from the main analysis.

#### Procedural memory test

To test the participants’ procedural memory, we used a non-verbal task based on Abla and Okanoya’s (2009) shape sequence test for statistical learning. The task consisted of two phases: a familiarization phase and a test phase. During the familiarization phase, the participants saw 300 pictures of white shapes against the black background, displaying one of the 12 shapes that were used in the task. The shape display was accompanied by background music. Four three-shape sequences, each with its unique set of shapes, were continuously played 25 times each, but the order in which this happened was randomized. All shapes were visible for 600 ms, and it took 3 min of viewing time to complete this part of the test.

During the test phase, which was administered immediately after the familiarization phase, 16 shape sequences were shown using a PowerPoint presentation, one white shape on a black background at a time. The shape sequences that were tested included all four three-shape sequences that had appeared in the familiarization phase and four sequences that were off by one symbol in the middle. The participants were asked to indicate on a response sheet whether a sequence at hand was familiar from the familiarization phase. The shape sequences in the testing phase were all shown twice, making a total of 16 shape sequences, and were shown in randomized order.

The participants were tested in small groups of max. 10 children at a time. The test materials were projected to a large screen in the classroom. Each correct response was scored 1 point, with a maximum test score of 16.

#### Verbal working memory test

Verbal working memory was measured with the Monkey Game (Van de Weijer-Bergsma et al., 2016), a computerized backward word span task for self-reliant administration that was developed for Dutch primary school children. A selection of nine simple Dutch words that are usually learnt in kindergarten were used in the task; namely, *maan* ‘moon’, *vis* ‘fish’, *roos* ‘rose’, *oog* ‘eye’, *huis* ‘house’, *ijs* ‘ice’, *vuur* ‘fire’, *poes* ‘cat’, and *jas* ‘coat’. The task consisted of five levels, which increased in difficulty. At level 1, only two words had to be remembered, while at level 5 six words had to be remembered. At each level, there were four trials with sequences that had to be remembered, so there were 20 trials in total. The Monkey Game has two response formats. One includes pictures of the sounded words in a 3 × 3 matrix, originally designed for grade 1, while the other includes the written version of those words, designed for grades 2–6. However, because children with DLD have a disadvantage when it comes to reading (Snowling et al., 2020), we opted for the matrix with pictures.

The participants performed the task individually on a laptop or computer. The test started with 2 practice trials in which participants had to recall 2 words forwards, after which 4 more sets of two-word practice trials were given in which the words had to be recalled backwards. Participants were asked to click on the pictures in the matrix in backwards order. They received feedback on the correctness of their response immediately after answering the practice trials. After that, the main test started during which they received no feedback on their performance. Most participants finished the test within 10 min.

Each time an item was clicked on in the right order, a point was scored. Thus, a point was given per correct item and not per correct sequence. If, for example, the pictures of moon, rose, fish and coat had to be clicked on, but rose, house, coat and house were filled in, the sequence was not correct, but the participant still scored two points because the response included two items that were in the correct position in the sequence. A maximum of 80 points could be scored for this task.

#### English out-of-school exposure questionnaire

To control for informal out-of-school exposure to English, we used the exposure questionnaire developed by Tribushinina and Mackaaij (2023). We used a child questionnaire rather than a parental questionnaire for several reasons. First, teenagers may know better than their parents how much time they spend watching English-spoken videos, which is the most important source of English exposure in the Dutch context (Tribushinina and Mackaaij, 2023). Second, many children with DLD have parents with DLD who prefer not to engage with textual materials such as questionnaires. Finally, as explained above, this research was conducted during the Covid pandemic when the parents were not allowed on the school premises. The use of a child questionnaire allowed us to collect exposure data from all participants.

The questionnaire was in Dutch and included four multiple-choice questions about frequency and four multiple-choice questions about duration of exposure to English with respect to listening (to music), watching (films, series, and videos), playing games, and reading (books, cartoons, magazines, blogs). These are the most common categories of out-of-school exposure to English identified in prior research (Sundqvist, 2009; Lindgren and Muñoz, 2013; Peters, 2018; Muñoz, 2020; Leona et al., 2021). The questions were formulated in a way that would allow primary school children with DLD to understand the questions and to give accurate estimates. For example, multiple descriptive options with frequency (e.g., *every day, a few time a week*) and duration adverbials (e.g., *all day long, a few hours a day*) were used instead of hour indications (How many hours a day do you…?) because children of this age may not yet have an accurate representation of time in hours and minutes. Previous studies using the questionnaire (Tribushinina and Mackaaij, 2023; Tribushinina et al., 2023) confirmed that 10- to 12-year-old children with DLD cope with the help of a researcher, and the provided estimates strongly correlate with various measures of performance in English.

For children with DLD, the questionnaire was administered individually so that a researcher could help them understand the questions and fill in the answers. The participants with TLD filled in the questionnaire independently, but the researchers could help them in case any difficulties arose.

The answers were translated to a Likert-scale, which ranged between 0 to 4 for the frequency questions, and 0 to 3 for the duration questions (see Tribushinina and Mackaaij, 2023 for a detailed scoring scheme). This led to a score ranging from 0 to 28.

### Statistical analyses

Data were analyzed using multilevel linear regression using the lmerTest package in R (Kuznetsova et al., 2017). Since participants sharing the same classroom (and hence teachers) and participants from the same schools are likely to have more in common than participants from different classes and schools (Hox et al., 2017), Class nested in School was included as a random effect in all the analyses. Story (Cat; Dog) was included in the random part of the models for the analyses of the narrative tasks. The scores on the Dutch and the English language tests constituted the outcome variables. For the analyses of the Dutch data, the predictor variables were Background (multilingual; monolingual), Group (DLD; TLD) and the interaction between Group and Background; the control variables were Age, Verbal Working Memory, Declarative Memory and Procedural Memory. For the analyses of the English data, the predictor variables were Background (multilingual; monolingual), Group (DLD; TLD) and their interaction; the control variables were Length of Instruction, Exposure, Age, Verbal Working Memory, Declarative Memory and Procedural Memory. Treatment coding was used in all the analyses, with the monolingual group and the DLD group as the reference levels. Below we only report significant results. Full model outputs can be found in Supplementary materials.

## Results

### Background variables

Table 2 presents the descriptive statistics for the background variables.

In the DLD group, monolinguals had higher declarative memory scores than multilinguals [*t*(35) = 2.31, *p* = 0.027]. There were no other significant differences between multilinguals and monolinguals. The only significant difference between children with and without DLD was that children with TLD had higher verbal working memory scores than their peers with DLD [*M*_TLD_ = 43.95, *M*_DLD_ = 34.95, *t*(73) = −3.82, *p* < 0.001].

### Correlations

Pearson correlations between all linguistic measures are presented in Table 3 for the two groups separately.

**Table 3:** Bivariate correlations among all language measures, by Group.

	1	2	3	4	5	6	7	8	9	10	11
Dutch											
1. SRT	–	*−0.10*	*−0.22*	*−0.10*	***−0.36***	*0.12*	*−0.25*	*−0.09*	*0.04*	*0.05*	*−0.12*
2. MLCU	**0.36**	–	***0.43***	*0.31*	***−0.35***	*−0.18*	*−0.07*	*0.20*	*−0.10*	*−0.12*	*0.14*
3. N word types	**0.45**	**0.57**	*–*	***0.90***	*−0.31*	*−0.09*	*−0.09*	*0.004*	*0.16*	*0.23*	*0.01*
4. N word tokens	0.**45**	**0.57**	**0.92**	–	***−0.35***	*−0.06*	*0.00*	*0.04*	*0.21*	*0.31*	*−0.06*
5. Error rate	**−0.37**	−0.23	**−0.47**	**−0.50**	–	*0.13*	*0.11*	*0.21*	*0.13*	*0.03*	*0.03*
English											
6. Vocabulary	−0.18	0.13	0.27	0.17	−0.00	–	***0.87***	***0.76***	***0.80***	***0.73***	*−0.32*
7. Grammar	−0.21	0.13	0.25	0.19	0.01	**0.86**	–	***0.76***	***0.77***	***0.73***	***−0.38***
8. MLCU	−0.00	0.25	**0.39**	0.29	−0.06	**0.73**	**0.76**	–	***0.90***	***0.83***	***−0.47***
9. N word types	0.01	0.22	0.**41**	0.26	−0.05	**0.77**	**0.76**	**0.89**	–	***0.95***	***−0.40***
10. N word tokens	0.03	0.24	**0.52**	**0.39**	−0.08	**0.73**	**0.72**	**0.88**	**0.94**	–	***−0.45***
11. Error rate	−0.14	−0.24	**−0.44**	**−0.68**	0.08	**−0.45**	**−0.61**	**−0.75**	**−0.64**	**−0.68**	–

Correlation coefficients for children with DLD are presented in the upper triangle (in italics); correlation coefficients for the TLD group are presented in the lower triangle. Significant correlations (*p* < 0.05) are in boldface. SRT, Sentence Repetition Task; MLCU, mean length of C-unit.

All correlations were positive, except the correlations between the language scores and the rate of grammatical errors in the narratives. Within-language correlations were stronger and more numerous than between-language correlations. In English, all the measures showed strong correlations with all other English measures, except the correlation between vocabulary task and error rates in the DLD group. In Dutch, most measures showed moderate to strong correlations with at least one other Dutch measure; the number of significant intra-language correlations was higher in the TLD group. In the TLD group, there were six significant cross-language correlations: Dutch lexical diversity was correlated with English syntactic complexity, lexical diversity, fluency and accuracy, Dutch fluency was significantly correlated with English fluency and accuracy. In the DLD group, none of the cross-language correlations were significant.

Pearson correlations between all memory measures are presented in Table 4. The only significant correlation was between verbal working memory and declarative memory in the TLD group.

**Table 4:** Bivariate correlations among the memory measures, by Group.

	1	2	3
1. Procedural memory	*–*	*0.31*	*0.32*
2. Declarative memory	0.30	–	*−0.02*
3. Verbal working memory	0.16	**0.58**	–

Correlation coefficients for children with DLD are presented in the upper triangle (in italics); correlation coefficients for the TLD group are presented in the lower triangle. Significant correlations (*p* < 0.05) are in boldface.

### Dutch measures

The means (and standard deviations) of the Dutch measures are presented in Table 5.

**Table 5:** Means (and standard deviations) of the Dutch measures.

	DLD		TLD	
	Multilingual	Monolingual	Multilingual	Monolingual
SRT (max. 90)	51.15 (14.82)	66.67 (6.99)	81.44 (6.99)	84.35 (5.10)
MAIN: MLCU	7.48 (1.17)	7.30 (1.01)	7.68 (1.09)	7.77 (1.41)
MAIN: N word types	43.00 (10.23)	37.39 (12.47)	41.79 (9.61)	41.15 (13.68)
MAIN: N word tokens	98.54 (28.16)	93.96 (24.81)	76.56 (31.97)	86.55 (33.50)
MAIN: error rate	0.12 (0.05)	0.09 (0.04)	0.10 (0.05)	0.06 (0.04)

SRT, Sentence Repetition Task; MAIN, Multilingual Assessment Instrument for Narratives; MLCU, mean length of C-unit.

The results of the multilevel linear regression analysis of the SRT data revealed that children with TLD outperformed their peers with DLD (*B =* 18.74*, SE* = 6.17, *t* = 3.04, *p* = 0.011). Multilingual children performed significantly worse than monolingual children (*B =* −7.94*, SE* = 3.48, *t* = −2.28, *p =* 0.026). But the interaction between Group and Background was not significant. The entire model (including the random part) explained 70% of the variance.

Error rates in the Dutch narratives were negatively predicted by age, with older children making fewer errors (*B =* −0.00*, SE* = 0.00, *t* = −2.01, *p* = 0.049). The multilinguals made significantly more errors than monolinguals (*B =* 0.04*, SE* = 0.02, *t* = 2.11, *p* = 0.039). Children with TLD made fewer errors than children with DLD (*B =* −0.03*, SE* = 0.02, *t* = −2.17, *p* = 0.034). The model explained 21% of the variance. For the other narrative measures, none of the predictors were significant. The interaction between Group and Background was not significant in any of the analyses (see full models in Supplementary materials).

### English measures

The descriptive statistics for the English measures are presented by group in Table 6.

**Table 6:** Means (and standard deviations) of the English measures.

	DLD		TLD	
	Multilingual	Monolingual	Multilingual	Monolingual
Vocabulary (max. 40)	20.08 (8.85)	13.42 (8.58)	26.00 (8.85)	20.40 (8.22)
Grammar (max. 30)	15.23 (6.10)	10.42 (4.10)	19.67 (5.47)	14.10 (5.19)
MAIN: MLCU	4.98 (1.66)	2.75 (1.45)	5.41 (1.95)	3.70 (2.05)
MAIN: N word types	21.38 (11.10)	8.58 (10.57)	23.94 (12.73)	13.75 (11.92)
MAIN: N word tokens	56.23 (34.27)	26.42 (23.62)	52.39 (27.61)	37.20 (30.81)
MAIN: error rate	0.26 (0.14)	0.24 (0.12)	0.19 (0.11)	0.25 (0.16)

MAIN, Multilingual Assessment Instrument for Narratives; MLCU, mean length of C-unit.

The outcomes of the vocabulary test were positively predicted by amount of exposure to English outside of the classroom (*B =* 0.78*, SE* = 0.16, *t* = 4.78, *p* < 0.001) and verbal working memory (*B =* 0.17*, SE* = 0.08, *t* = 2.06, *p* = 0.044), but negatively by procedural memory scores (*B =* −0.84*, SE* = 0.36, *t* = −2.32, *p* = 0.023). Multilingual children outperformed their monolingual peers (*B =* 6.74*, SE* = 2.50, *t* = 2.70, *p* = 0.009). The effect of Group and the interaction between Group and Background were not significant. The model explained 56% of the variance.

The performance on the grammar test, syntactic complexity of narratives (MLCU) and lexical diversity of narratives (N types) were positively predicted by multilingual status (*B_GRAM_ =* 4.36*, SE_GRAM_* = 1.65, *t_GRAM_* = 2.64, *p_GRAM_* = 0.011; *B_MLCU_ =* 2.24*, SE_MLCU_* = 0.56, *t_MLCU_* = 3.98, *p_MLCU_* < 0.001; *B_NTYPES_ =* 13.10*, SE_NTYPES_* = 3.51, *t_NTYPES_* = 3.73, *p_NTYPES_* < 0.001) and out-of-school exposure to English (*B_GRAM_ =* 0.47*, SE_GRAM_* = 0.11, *t_GRAM_* = 4.50, *p_GRAM_* < 0.001; *B_MLCU_ =* 0.17*, SE_MLCU_* = 0.04, *t_MLCU_* = 4.78, *p_MLCU_* < 0.001; *B_NTYPES_ =* 1.05*, SE_NTYPES_* = 0.23, *t_NTYPES_* = 4.61, *p_NTYPES_* < 0.001). None of the interactions were significant. The models explained 59% (grammar), 51% (MLCU) and 52% (N types) of the variance.

The results for the fluency of narratives (N tokens) demonstrate that the bilingual children with DLD produced longer narratives than the monolingual children with DLD (*B =* 33.45*, SE* = 8.96, *t* = 3.73, *p* < 0.001). However, this difference was not found in the TLD group, as evidenced by the significant interaction between Group and Background (*B =* −28.72*, SE* = 12.92, *t* = −2.22, *p* = 0.030). Exposure positively predicted the number of words used in a narrative (*B =* 2.30*, SE* = 0.58, *t* = 3.94, *p* < 0.001). This model explained 44% of the variance.

For the accuracy of the English narratives, only exposure to English outside of the classroom was associated with lower error rates (*B =* −0.01*, SE* = 0.00, *t* = −2.79, *p* = 0.007). This model explained 17% of the variance.

## Discussion

The study by Tribushinina and Mackaaij (2023) was the first to demonstrate that multilingual children with DLD tend to outperform their monolingual peers with DLD on EFL skills. The present study set out to replicate this finding and to test the hypothesis that the effects of multilingualism on FL learning would be stronger in multilinguals with TLD compared to their peers with DLD because DLD may negatively affect three of the four potential sources of multilingual adaptations discussed in the literature (transfer, executive functions, and metalinguistic awareness). Therefore, we adopted a 2 × 2 design and compared the performance of monolinguals and multilinguals with and without DLD. Although our main focus was on EFL, we also compared the performance of these four groups in Dutch (majority/school language), as previous studies indicate that the same multilinguals who demonstrate enhanced performance in English may be outperformed by their monolingual peers on proficiency in the ML (see Hopp et al., 2019 for TLD and Tribushinina and Mackaaij, 2023 for DLD). Finally, we included a number of control variables that were deemed relevant for studying individual differences and multilingual effects in EFL performance. We discuss the findings for English first, followed by the discussion of the Dutch results and the findings on the background variables.

### Positive effects of multilingualism in EFL

The findings revealed that 9- to 12-year-old multilinguals outperformed their monolingual peers on English vocabulary, grammar and oral skills measured by lexical diversity, syntactic complexity and fluency of narratives. The only measure revealing no differences between monolinguals and multilinguals was grammatical accuracy of narratives. These results replicate the findings reported by Tribushinina and Mackaaij (2023) for pupils with DLD of the same age range. In fact, the effect of multilingualism was even more robust in the present study. Tribushinina and Mackaaij (2023) also found differences between multilinguals and monolinguals on vocabulary, grammar and syntactic complexity of narratives. However, in their study the positive effect of multilingualism on lexical diversity and fluency of narratives became non-significant after controlling for differences in out-of-school exposure to English. In contrast, in our study, the group differences still hold after controlling for exposure and cognitive skills. The converging evidence from our current study and Tribushinina and Mackaaij (2023) runs counter to the proposal that multilingual effects in FL learning may be limited to the lexical level (Aguasvivas et al., 2024), as both studies report enhanced performance of the multilingual groups on English grammar. The current results suggest that the positive effects of multilingualism on FL learning, often attested in typically-developing pupils (Cenoz, 2003; Hirosh and Degani, 2018; Festman, 2021), also hold for vulnerable language learners with language disorders.

Even more importantly, we found no evidence that the effect of multilingualism on EFL performance would be stronger in pupils without DLD. The interaction between Group and Background was only significant for fluency in the narrative task, but the interaction was in the opposite direction to the one we predicted: The difference between multilinguals and monolinguals was only attested in the DLD group. So, contrary to our hypothesis, there is no evidence that positive effects of multilingualism on English skills would be more pronounced in children with TLD.

It might be the case that our initial assumption that DLD negatively affects positive cross-language transfer and metalinguistic awareness does not hold. Recall that evidence so far is extremely limited and mixed. Furthermore, even if metalinguistic awareness is negatively affected by the disorder, in the sense that children with DLD may have lower scores than peers with TLD (Kamhi and Koenig, 1985), multilingual children with DLD might still be more metalinguistically aware than their monolingual peers with DLD (cf. Peristeri et al., 2019). Our study did not include a measure of metalinguistic awareness. However, the results reported in Tribushinina and Mackaaij (2023) are informative for interpreting our results. In their study, bilinguals with DLD outperformed monolinguals with DLD on the English grammar test (language proficiency measure), but not on the English grammaticality judgment task (a metalinguistic measure). More research comparing monolinguals and multilinguals with DLD on metalinguistic skills, using comparable measures of metalinguistic awareness, is urgently needed. Future research on this topic will also benefit from studying the mediating role of metalinguistic awareness in explaining the differences between multilinguals and monolinguals with DLD on FL skills (cf. Rauch et al., 2011).

Regarding the availability of transfer, we based our prediction on the scarce results suggesting that positive transfer of grammar knowledge is less/not available to L2/EFL learners with DLD (Ebert et al., 2014; Blom and Paradis, 2015; Tribushinina et al., 2020). At the same time, there is more recent evidence that grammar skills in the ML (Dutch) significantly predict grammar skills in EFL in a sample of primary school children with DLD (Stolvoort et al., 2023), which might be taken as evidence of transferability of grammar knowledge and/or skills between languages (see also Blom and Paradis, 2013). In the L2 literature, significant cross-language relations have often been taken as evidence of linguistic interdependence and positive transfer from L1 to L2 (e.g., Verhoeven et al., 2012; Siu and Ho, 2015; Tribushinina et al., 2020; Blom et al., 2021; Lorenz et al., 2024; Stolvoort et al., 2023). If significant inter-language correlations can indeed be seen as tokens of positive transfer, our results are more in line with the literature suggesting that cross-language transfer is less available to children with DLD: There were six significant correlations between Dutch and English in the TLD group and none in the DLD sample. If the cross-language correlations were merely a reflection of the instruction language effects, we would expect to find such correlations in both groups. However, in the absence of HL data, we cannot make any firm conclusions in this regard.

Studying the workings of positive transfer as an underlying mechanism of multilingual effects on novel language learning was beyond the scope of our research. To understand the underlying mechanisms of multilingual adaptations in FL learning, more research into the influence of DLD on cross-language transfer (particularly in the domain of grammar) is needed. It is also important to keep in mind that correlations present only indirect evidence of positive transfer because these relationships can also be due to a third overarching factor. Furthermore, we did not take the properties of the HLs spoken by the multilinguals into account (due to a mixed sample). To gain more insights into the workings of positive transfer in DLD, future research will benefit from studies investigating transfer directly, based on the typological properties of L1, L2 and L3. For example, our recent study on the acquisition of L3 English aspect by Dutch primary-school pupils with TLD has shown that the narratives of Serbian-Dutch bilinguals show traces of positive transfer from Serbian and negative transfer from Dutch (Tribushinina et al., 2024b), which suggests that property-by-property transfer is not limited to the most typologically similar language (cf. Westergaard et al., 2017; Kolb et al., 2022). It appears more challenging to sample homogeneous multilingual groups in clinical populations. To the best of our knowledge, only two studies have compared the role of L1 typology in L2 acquisition by children with and without DLD, and these two studies provided conflicting findings on the availability of L1 transfer in DLD (Blom and Paradis, 2013, 2015). Studies directly testing the presence and contribution of positive transfer to the multilingual EFL effect across populations are sorely needed.

In this study, we controlled for declarative memory, procedural memory and verbal working memory because these memory systems have been shown to play an important part in FL learning (e.g., Baddeley, 2003; Kormos and Sáfár, 2008; Engel de Abreu and Gathercole, 2012; Verhagen and Leseman, 2016) and because multilingualism may have a positive effect on these memory systems (e.g., de Jesus, 2012; Blom et al., 2014; Wang and Saffran, 2014; Poepsel and Weiss, 2016; Monnier et al., 2022). However, the strongest effects of multilingualism on cognitive skills in children have been reported for selective attention, interference suppression and cognitive flexibility (see Giovannoli et al., 2020 for a review), which we did not control for. So far, only one study comparing monolinguals and multilinguals on (E)FL skills controlled for differences in both verbal working memory (measured with forward and backward digit span tasks) and executive control (measured with the Simon Task) (Hopp et al., 2019). Working memory was found to predict EFL performance in productive vocabulary in grade 3 and receptive grammar in grade 4 (similar to our findings for vocabulary), whereas executive control did not predict EFL performance. Nevertheless, it is still possible that differences in executive control could mediate the relationship between multilingualism and FL performance, for example, because children with enhanced attentional control attend more to cues in the input (Blom et al., 2017; Poepsel and Weiss, 2016; Pons et al., 2015) and have enhanced control of attention to conflicting cues (Blom et al., 2014; Carlson and Meltzoff, 2008; Engel de Abreu et al., 2012; Kroll and Bialystok, 2013). Therefore, we recommend that future studies should test a mediating role of selective attention, interference suppression and cognitive flexibility in explaining the effects of multilingualism on novel language learning by children with and without DLD. We are currently exploring these possibilities in our lab.

### Lack of DLD effects in EFL

Our main aim was to compare the effects of multilingualism on EFL learning *within* groups (DLD and TLD) and to compare the extent of such effects in the two groups. Although it was not our aim to compare EFL performance of children with and without DLD directly, it is still worth mentioning the surprising finding that pupils with DLD performed on a par with their typically-developing peers in all English skills measured in this study, despite having poorer verbal working memory. Only in the case of the English vocabulary test, the difference between groups approached significance (*p = 0*.088). This result might be due to the fact that participants with DLD in special education and participants with TLD in mainstream education received different kinds of EFL instruction. In mainstream primary schools in the Netherlands, English is taught following a communicative approach, not including explicit grammar instruction. Our participants with DLD received English lessons following a multisensorial explicit method specifically designed for pupils with DLD (Tribushinina et al., 2022). Explicit teaching approaches have been shown to neutralize individual differences in language aptitude (Erlam, 2005) and may have thus been beneficial for the participants with DLD. Similarly, a study conducted with Russian-speaking pupils with DLD showed that after 1 year of English lessons following an explicit teaching approach, there were no significant differences in EFL vocabulary and grammar between pupils with and without DLD (Tribushinina et al., 2020). However, after 2 years of instruction participants with TLD outperformed their peers with DLD. Since the participants in the present study had just started their English lessons at school, it is plausible that children with TLD will eventually show steeper progress.

### Negative effects of multilingualism and DLD in the majority language

We predicted that both multilingualism and DLD would be associated with lower test scores in the ML (Dutch), albeit for different reasons. This hypothesis was partly supported by our data. We found no effects of multilingualism and DLD in narrative fluency, lexical diversity and syntactic complexity, but both multilingualism and DLD negatively affected grammatical accuracy of narratives and performance on the sentence repetition task. The multilingual DLD group obtained the lowest scores of the four groups, replicating prior research on the effects of DLD in multilingual children in the Netherlands (Orgassa and Weerman, 2008; Verhoeven et al., 2011). The Litmus SRT was designed for the diagnostics of DLD in multilingual populations (Marinis and Armon-Lotem, 2015). Our results confirm that this measure is sensitive to the effects of both reduced exposure (in multilingualism) and reduced intake (in DLD). This task has usually been used with younger children (up to 8 years of age). Our findings reveal that the task is also suitable for children up to age 13. It is interesting to observe that the effects of multilingualism on ML performance may persist into pre-adolescence.

Despite the lower performance of multilinguals in the ML, our results are in line with previous research demonstrating that multilingualism does not form an additional disadvantage to children with DLD (e.g., Paradis et al., 2006; Paradis, 2007; Armon-Lotem, 2010; Blom and Boerma, 2017). According to the cumulative effects hypothesis (Paradis, 2010; Paradis et al., 2017), the gap between multilingual and monolingual children should be larger in the DLD group. However, the interactions between multilingual status and clinical status were not significant in our study, providing no evidence of cumulative effects (cf. Novogrodsky and Meir, 2020).

Our hypothesis that monolinguals would outperform multilinguals on ML skills was based on ample research showing that reduced ML exposure in multilinguals contexts may negatively affect ML proficiency. However, our study did not include a measure of ML exposure, which is a limitation. Furthermore, even though we recruited monolinguals and multilinguals in the same schools (and schools in the Netherlands are relatively homogenous in terms of SES, see Sykes and Musterd, 2011), we could not control for SES effects at the individual level. It is possible that multilinguals had a lower SES than monolinguals, which also played a role in their poorer performance in Dutch. However, one of the most interesting findings is that the same multilinguals outperformed their monolingual peers in English. Our findings confirm the results of the previous studies that found an intriguing asymmetry between FL and ML skills in children with TLD (Hopp et al., 2019) and DLD (Tribushinina and Mackaaij, 2023). This asymmetry is remarkable because ML skills are known to predict EFL skills, particularly if the languages are typologically close, as in the case of Dutch and English (Tribushinina et al., 2021, 2024a) and German and English (Edele et al., 2018; Lorenz et al., 2020, 2024). Indeed, we found several significant positive correlations between Dutch and English in the TLD sample. Children with higher scores in Dutch are also likely to have higher scores in English, which might be due to typological similarity or because Dutch was the language of instruction. Since multilingual children in our study (and also in Hopp et al., 2019; Tribushinina and Mackaaij, 2023) had lower scores than monolinguals on some of the tasks in Dutch, we would also expect them to have lower scores in English. However, the pattern in EFL is reversed, with multilinguals outperforming monolinguals on almost all measures. This intriguing pattern suggests that the sources of multilingual effects in EFL learning should probably be sought in non-linguistic domains, such as executive functioning and language learning attitudes.

### Background variables

The only background variable that significantly contributed to all measures of English proficiency included in this study (vocabulary, grammar and narrative fluency, accuracy, syntactic complexity and lexical diversity) was out-of-school exposure to English. This result replicates previous research demonstrating that out-of-school exposure is one of the strongest predictors of EFL proficiency (Lindgren and Muñoz, 2013; Sundqvist and Sylvén, 2016; De Wilde and Eyckmans, 2017; Puimège and Peters, 2019; De Wilde et al., 2020; Leona et al., 2021). Interestingly, length of English instruction was not a significant predictor in any of the models. This finding is consistent with previous studies conducted in European countries in which children have ample exposure to English outside of the classroom. For example, Peters (2018) found that formal EFL instruction explained 7% of variance in EFL vocabulary outcomes in Flemish adolescents in Belgium, whereas amount of out-of-school exposure explained 13% of variance. In the Netherlands, Leona et al. (2021) compared English vocabulary skills of primary school children who had received several years of English instruction and their peers not yet receiving lessons in English. Remarkably, no differences were found between the two groups. Similarly, Tribushinina et al. (2024a) report that out-of-school exposure to English significantly predicted English vocabulary and grammar scores in a sample of monolingual and bilingual secondary school pupils in the Netherlands, whereas amount and length of formal EFL instruction did not explain performance on any of the tests. This is probably due to the fact that in countries where children have extensive out-of-school exposure to English, the amount of classroom time is too limited to make a difference.

Unlike prior studies comparing monolingual and multilingual pupils on the amount of out-of-school exposure to English, we did not find group differences in the amount of extramural English. The previous studies have shown that multilingual children growing up in expat communities may have more exposure to English than their monolingual peers (Tribushinina and Mackaaij, 2023). At the same time, multilinguals belonging to large minority groups, such as the Turkish community in the Netherlands, have been shown to have less experience with English outside of the classroom (Tribushinina et al., 2024a). In our study comparing monolinguals to a mixed multilingual sample, no group differences emerged. Nevertheless, since out-of-school exposure significantly predicted all English skills measured in this study, we recommend including measures of extramural English in all studies comparing monolingual and multilingual groups on FL (particularly English) outcomes.

In this study, we also controlled for verbal working memory, declarative memory and procedural memory because these memory systems have been shown to be sensitive to multilingual effects and to play an important part in FL learning. We did not find positive effects of multilingualism on any of the memory measures, confirming that the effects of multilingualism on cognitive skills are elusive and controversial (Duñabeitia et al., 2014; Paap et al., 2015; Bulgarelli et al., 2018; Giovannoli et al., 2020). The only group difference that we found was in the opposite direction, with monolinguals with DLD outperforming multilinguals with DLD on declarative memory. This difference cannot be attributed to smaller vocabularies or poorer Dutch skills in the multilingual sample because we used a non-verbal declarative memory task. Although cognitive adaptations in multilingual children have been reported in numerous studies, it still needs to be established under what circumstances group differences in cognitive skills may arise (e.g., with which tasks) and in what multilingual groups (Leivada et al., 2021; Festman et al., 2023; Masullo et al., 2024). A limitation of our study is that for practical reasons (time pressure on parents and teachers during the Covid pandemic) we could not collect information on the type of multilingualism (simultaneous or sequential), age of L2 onset, HL use and proficiency. Future studies collecting such information can provide valuable insights into what aspects of multilingual experience may lead to linguistic and cognitive adaptations.

Surprisingly, the performance on the declarative memory task did not predict any of the English (or Dutch) measures included in this study. Declarative memory is considered to play an important role in vocabulary learning (Ullman and Pierpont, 2005) and has also been shown to be involved in the acquisition of grammar by children with DLD (Lum et al., 2012). Procedural memory only explained the performance in the English vocabulary test, but the relationship was negative. Since declarative and procedural memory scores were not correlated in either group, it is difficult to explain this unexpected result. But it should be noted that Tagarelli et al. (2016) also reported a negative relationship between procedural memory and artificial language learning under incidental learning conditions, which they could not explain. English vocabulary scores were positively predicted by verbal working memory, which is in line with prior research showing that verbal working memory is involved in FL learning (Baddeley, 2003; Engel de Abreu and Gathercole, 2012; Tagarelli et al., 2016) and in learning FL vocabulary in particular (Kormos and Sáfár, 2008).

We found no differences between children with and without DLD on the declarative memory and procedural memory task. The former finding is not surprising assuming that declarative memory is spared in DLD (Ullman and Pierpont, 2005; Lum et al., 2012). However, the fact that we found no differences in the procedural memory task is surprising assuming that DLD is associated with a procedural learning deficit (Ullman and Pierpont, 2005). It might be the case that our procedural memory task was not sensitive enough. At the same time, there have been other studies that found no effect of DLD on visual (non-verbal) procedural learning (see, for example, a meta-analysis and an experimental study reported in Lammertink et al., 2020). It is possible that procedural learning disadvantages in DLD are more likely to be pinpointed in linguistic tasks, such as artificial mini-language learning paradigms. It is also possible that deficits in procedural memory can be explained by working memory limitations in DLD (Jackson et al., 2020). Recall that in our study, children with DLD had weaker working memory. More research on the impact of DLD on procedural memory, in relation to FL learning, is needed.

## Conclusion

This study demonstrated that multilingual children with and without DLD outperform their monolingual peers on English skills, confirming that multilingualism may have a positive effect on novel language learning. Interestingly, the interaction between Group and Background was not significant. So, there are no reasons to assume that multilingual effects are stronger in typically-developing children. Our findings also confirm that out-of-school exposure to English is an important predictor of English outcomes in pupils with TLD and DLD. DLD had a negative effect on verbal working memory, but not on declarative or procedural memory, and not on proficiency in English. Finally, both DLD and multilingualism negatively predicted performance on the Sentence Repetition Task and on the accuracy of narratives in the majority language, but there were no cumulative disadvantages.

## Data Availability

The raw data supporting the conclusions of this article will be made available by the authors, without undue reservation.
