# Supplementary material for: The effects of heritage multilingualism on foreign language learning: a comparison of children with typical language development and developmental language disorder
Source: Front Psychol. 2025 Jan 15;15:1521340. doi: 10.3389/fpsyg.2024.1521340 (PMC11774774; doi:10.3389/fpsyg.2024.1521340)
Supplement: Supplementary file 1 [file Table_1.docx]

Table S1. Coefficients of the comparisons between groups on the Dutch SRT

|  | *B* | *SE* | *t* value | *p* value |
| --- | --- | --- | --- | --- |
| (Intercept) | 64.42 | 23.18 | 2.78 | .008 |
| Background (bi) | –7.94 | 3.48 | –2.28 | .026 |
| Group (TLD) | 18.74 | 6.17 | 3.04 | .011 |
| Age in Months | –0.17 | 0.16 | –1.08 | .284 |
| Procedural Memory | 0.76 | 0.50 | 1.50 | .137 |
| Declarative Memory | 0.35 | 0.33 | 1.06 | .295 |
| Verbal Working Memory | 0.07 | 0.12 | 0.60 | .552 |
| Group (TLD): Background (bi) | 6.11 | 5.80 | 1.05 | .297 |

Table S2. Coefficients of the comparisons between groups on Dutch syntactic complexity (MLCU in MAIN)

|  | *B* | *SE* | *t* value | *p* value |
| --- | --- | --- | --- | --- |
| (Intercept) | 8.09 | 2.65 | 3.05 | .004 |
| Background (bi) | 0.23 | 0.41 | 0.56 | .581 |
| Group (TLD) | 0.54 | 0.66 | 0.82 | .441 |
| Age in Months | –0.01 | 0.02 | –0.79 | .437 |
| Procedural Memory | –0.00 | 0.06 | –0.08 | .940 |
| Declarative Memory | 0.04 | 0.04 | 1.14 | .260 |
| Verbal Working Memory | –0.01 | 0.01 | –0.38 | .708 |
| Group (TLD): Background (bi) | –1.08 | 0.67 | –1.62 | .112 |

Table S3. Coefficients of the comparisons between groups on Dutch lexical diversity (N word types in MAIN)

|  | *B* | *SE* | *t* value | *p* value |
| --- | --- | --- | --- | --- |
| (Intercept) | –6.20 | 23.66 | –0.26 | .795 |
| Background (bi) | 2.15 | 4.02 | 0.53 | .595 |
| Group (TLD) | 3.63 | 5.36 | 0.68 | .514 |
| Age in Months | 0.25 | 0.16 | 1.53 | .137 |
| Procedural Memory | 0.11 | 0.57 | 0.20 | .846 |
| Declarative Memory | 0.40 | 0.37 | 1.07 | .286 |
| Verbal Working Memory | –0.00 | 0.14 | –0.04 | .972 |
| Group (TLD): Background (bi) | –9.47 | 6.13 | –1.55 | .129 |

Table S4. Coefficients of the comparisons between groups on Dutch fluency (N word tokens in MAIN)

|  | *B* | *SE* | *t* value | *p* value |
| --- | --- | --- | --- | --- |
| (Intercept) | –7.03 | 62.97 | –0.11 | .912 |
| Background (bi) | 5.31 | 10.02 | 0.53 | .598 |
| Group (TLD) | 5.11 | 15.35 | 0.33 | .747 |
| Age in Months | 0.36 | 0.43 | 0.85 | .401 |
| Procedural Memory | 0.39 | 1.44 | 0.27 | .786 |
| Declarative Memory | 1.56 | 0.94 | 1.66 | .101 |
| Verbal Working Memory | –0.25 | 0.34 | –0.72 | .472 |
| Group (TLD): Background (bi) | –17.36 | 15.94 | –1.09 | .282 |

Table S5. Coefficients of the comparisons between groups on Dutch accuracy (N error rates in MAIN)

|  | *B* | *SE* | *t* value | *p* value |
| --- | --- | --- | --- | --- |
| (Intercept) | 0.17 | 0.08 | 2.18 | .033 |
| Background (bi) | 0.04 | 0.02 | 2.11 | .039 |
| Group (TLD) | –0.03 | 0.02 | –2.17 | .034 |
| Age in Months | –0.00 | 0.00 | –2.01 | .049 |
| Procedural Memory | 0.00 | 0.00 | 0.76 | .451 |
| Declarative Memory | 0.00 | 0.00 | 0.73 | .469 |
| Verbal Working Memory | 0.00 | 0.00 | 0.18 | .860 |
| Group (TLD): Background (bi) | 0.01 | 0.02 | 0.25 | .807 |

Table S6. Coefficients of the comparisons between groups on the English vocabulary task

|  | *B* | *SE* | *t* value | *p* value |
| --- | --- | --- | --- | --- |
| (Intercept) | 9.48 | 14.61 | 0.65 | .519 |
| Background (bi) | 6.74 | 2.50 | 2.70 | .009 |
| Group (TLD) | 4.37 | 2.53 | 1.73 | .088 |
| Exposure | 0.78 | 0.16 | 4.78 | < .001 |
| Length of EFL Instruction | 0.14 | 0.10 | 1.35 | .181 |
| Age in Months | 0.02 | 0.11 | 0.14 | .887 |
| Procedural Memory | –0.84 | 0.36 | –2.32 | .023 |
| Declarative Memory | –0.22 | 0.23 | –0.96 | .342 |
| Verbal Working Memory | 0.17 | 0.08 | 2.06 | .044 |
| Group (TLD): Background (bi) | –1.92 | 3.60 | –0.53 | .595 |

Table S7. Coefficients of the comparisons between groups on the English grammar task

|  | *B* | *SE* | *t* value | *p* value |
| --- | --- | --- | --- | --- |
| (Intercept) | 5.17 | 10.36 | 0.50 | .620 |
| Background (bi) | 4.36 | 1.65 | 2.64 | .011 |
| Group (TLD) | 2.62 | 2.16 | 1.22 | .265 |
| Exposure | 0.47 | 0.11 | 4.50 | < .001 |
| Length of EFL Instruction | –0.00 | 0.08 | –0.03 | .975 |
| Age in Months | 0.00 | 0.07 | 0.05 | .963 |
| Procedural Memory | –0.34 | 0.24 | –1.39 | .168 |
| Declarative Memory | –0.00 | 0.15 | –0.02 | .982 |
| Verbal Working Memory | 0.09 | 0.06 | 1.54 | .127 |
| Group (TLD): Background (bi) | –1.34 | 2.55 | –0.53 | .602 |

Table S8. Coefficients of the comparisons between groups on English syntactic complexity (MLCU in MAIN)

|  | *B* | *SE* | *t* value | *p* value |
| --- | --- | --- | --- | --- |
| (Intercept) | –1.65 | 3.29 | –0.50 | .617 |
| Background (bi) | 2.24 | 0.56 | 3.98 | < .001 |
| Group (TLD) | 0.66 | 0.57 | 1.16 | .252 |
| Exposure | 0.17 | 0.04 | 4.78 | < .001 |
| Length of EFL Instruction | 0.01 | 0.02 | 0.49 | .626 |
| Age in Months | 0.01 | 0.02 | 0.29 | .776 |
| Procedural Memory | 0.07 | 0.08 | 0.91 | .364 |
| Declarative Memory | –0.00 | 0.05 | –0.02 | .987 |
| Verbal Working Memory | 0.02 | 0.02 | 0.90 | .372 |
| Group (TLD): Background (bi) | –1.09 | 0.81 | –1.34 | .184 |

Table S9. Coefficients of the comparisons between groups on English lexical diversity (N word types in MAIN)

|  | *B* | *SE* | *t* value | *p* value |
| --- | --- | --- | --- | --- |
| (Intercept) | –44.12 | 20.56 | –2.15 | .036 |
| Background (bi) | 13.10 | 3.51 | 3.73 | < .001 |
| Group (TLD) | 5.34 | 3.56 | 1.50 | .138 |
| Exposure | 1.05 | 0.23 | 4.61 | < .001 |
| Length of EFL Instruction | –0.05 | 0.14 | –0.32 | .748 |
| Age in Months | 0.25 | 0.15 | 1.66 | .102 |
| Procedural Memory | 0.04 | 0.51 | 0.07 | .944 |
| Declarative Memory | 0.06 | 0.32 | 0.20 | .846 |
| Verbal Working Memory | 0.16 | 0.12 | 1.36 | .178 |
| Group (TLD): Background (bi) | –8.94 | 5.07 | –1.76 | .082 |

Table S10. Coefficients of the comparisons between groups on English fluency (N word tokens in MAIN)

|  | *B* | *SE* | *t* value | *p* value |
| --- | --- | --- | --- | --- |
| (Intercept) | –93.27 | 52.42 | –1.78 | .080 |
| Background (bi) | 33.45 | 8.96 | 3.73 | < .001 |
| Group (TLD) | 8.42 | 9.07 | 0.93 | .357 |
| Exposure | 2.30 | 0.58 | 3.94 | < .001 |
| Length of EFL Instruction | 0.01 | 0.37 | 0.03 | .973 |
| Age in Months | 0.40 | 0.38 | 1.03 | .305 |
| Procedural Memory | –0.45 | 1.30 | –0.35 | .728 |
| Declarative Memory | 0.75 | 0.81 | 0.93 | .356 |
| Verbal Working Memory | 0.49 | 0.30 | 1.64 | .106 |
| Group (TLD): Background (bi) | –28.72 | 12.92 | –2.22 | .030 |

Table S11. Coefficients of the comparisons between groups on English accuracy (error rates in MAIN)

|  | *B* | *SE* | *t* value | *p* value |
| --- | --- | --- | --- | --- |
| (Intercept) | 0.35 | 0.28 | 1.25 | .218 |
| Background (bi) | 0.01 | 0.05 | 0.20 | .842 |
| Group (TLD) | 0.03 | 0.05 | 0.65 | .517 |
| Exposure | –0.01 | 0.00 | –2.79 | .007 |
| Length of EFL Instruction | 0.00 | 0.00 | 0.05 | .962 |
| Age in Months | 0.00 | 0.00 | 0.81 | .423 |
| Procedural Memory | –0.00 | 0.01 | –0.66 | .509 |
| Declarative Memory | –0.00 | 0.00 | –1.06 | .293 |
| Verbal Working Memory | –0.00 | 0.00 | –0.15 | .879 |
| Group (TLD): Background (bi) | –0.05 | 0.07 | –0.79 | .435 |
